# Transforming ground mica into high-performance biomimetic polymeric mica film

**DOI:** 10.1038/s41467-018-05355-6

**Published:** 2018-07-30

**Authors:** Xiao-Feng Pan, Huai-Ling Gao, Yang Lu, Chun-Yan Wu, Ya-Dong Wu, Xiang-Ying Wang, Zhi-Qiang Pan, Liang Dong, Yong-Hong Song, Huai-Ping Cong, Shu-Hong Yu

**Affiliations:** 1 Key Laboratory of Advanced Catalytic Materials and Reaction Engineering, School of Chemistry and Chemical Engineering, Hefei University of Technology, Hefei, 230009 China; 2 Division of Nanomaterials & Chemistry, Hefei National Research Center for Physical Sciences at the Microscale, CAS Center for Excellence in Nano science, Hefei Science Center of CAS, Collaborative Innovation Center of Suzhou Nano Science and Technology, Hefei, 230026 China; 3 Department of Chemistry, University of Science and Technology of China, Hefei, 230026 China; 4 School of Electronic Science & Applied Physics, Hefei University of Technology, Hefei, 230009 China

## Abstract

Biomimetic assembly of high-quality nanosheets into nacre-like structures can produce macroscopic films with favorable mechanical and optical performances due to the intrinsic properties and high level of ordering of the nanoscale building blocks. Natural ground mica is abundant and exhibits great application potential. However, large size and low aspect ratio greatly limit its biomimetic assembly. Moreover, exfoliation of ground mica into high-quality nanosheets remains a significant challenge. Here, we report that large-scale exfoliation of ground mica into mono- or few-layered mica nanosheets with a production rate of ~1.0 g h^−^^1^ can be successfully achieved. The mica nanosheets are then assembled into strong biomimetic polymeric mica film that inherits the high electric insulation, excellent visible transmittance, and unique ultraviolet-shielding properties of natural mica. Its overall performance is superior to that of natural sheet mica and other biomimetic films, making the polymeric mica film a suitable substrate for flexible and transparent devices.

## Abstract (web summary)

Biomimetic assembly of nanosheets into nacre-like structures and films is of interest for a range of applications; the abundance of mica makes it a good candidate. Here, the authors report on the large-scale exfoliation of ground mica into nanosheets and the assembly into polymeric mica films.

## Introduction

Due to the excellent mechanical, optical, and electrical properties of ultrathin nanosheets, various kinds of layered materials have been successfully exfoliated into single or few layers^1–3^, including graphene^4^, hexagonal boron nitride^5^, transition metal dichalcogenides^6^, layered metal oxides (such as MnO_2_ and MoO_3_)^7^, black phosphorus^8^, layered double hydroxides (LDHs)^9^, and layered clays (such as montmorillonite, MTM). In particular, large-scale exfoliation of graphene and MTM has been successfully achieved^10,11^.

Mica, the only macroscopic transparent sheet-like natural layered clay, has attracted great attention due to its impressive properties, including visible-light transparency, ultraviolet (UV)-shielding, atomic level flatness, electric insulation, temperature stability, and chemical durability^12–15^. More recently, thin sheet mica with a macroscopic area and a thickness below 100 nm was mechanically exfoliated from natural sheet mica by Scotch tape. This thin sheet mica exhibits satisfactory performance when used as a gate insulator for organic field-effect transistors (OFETs)^16^ and as an electret in OFETs for multistate storage devices^17^. Though impressive results have been demonstrated in these areas, sheet mica is extremely rare, expensive, and brittle. In contrast, natural ground mica is more available and much cheaper and possesses no difference in chemical composition or layered structure relative to natural sheet mica^18–20^. Thus, it would be of great significance if natural ground mica could be assembled into large-size films with similar or superior performance relative to sheet mica.

Generally, biomimetic assembly of high-quality nanosheets into nacre-like “brick-and-mortar” structures is an efficient way to build high-performance macroscopic films due to the intrinsic properties and high level of ordering of nano-building blocks^10,21–27^. However, due to its large size and low aspect ratio, ground mica is not beneficial to be fabricated into high-performance macroscopic films via biomimetic assembly, and exfoliation of ground mica into high-quality mica nanosheets has always been a significant challenge and has rarely been reported^28^. As a result, in the highly attractive and valuable area of high-performance biomimetic composites, mica has not been well represented, while other clay minerals and 2D nanomaterials are frequently employed^29,30^.

Herein, we develop a facile and scalable exfoliating method to obtain high-quality mono-layered or few-layered mica nanosheets from natural ground mica with a production rate of ~1.0 g h^−1^. The exfoliated mica (eMica) nanosheets are then assembled with chitosan (CS) into macroscopic biomimetic polymeric mica films via a spray-coating method. It is found that the obtained polymeric mica film containing 60 wt.% of eMica nanosheets exhibits favorable mechanical properties, high electric insulation, superior visible transmittance, and unique UV-ageing resistance, all of which are highly desirable properties for flexible and transparent electronic devices.

## Results

### Large-scale exfoliation of natural ground mica

**Fig. 1:**
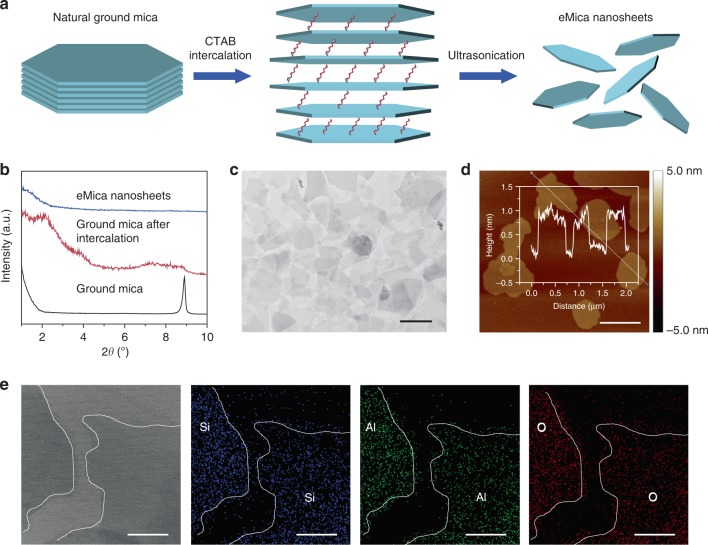
Characterization of eMica nanosheets. **a** Schematic illustration showing the exfoliation process of natural ground mica into eMica nanosheets via intercalation with CTAB followed by ultrasonication treatment. **b** Small-angle XRD patterns of ground mica, mica after intercalation, and eMica nanosheets. **c**, **d** TEM and AFM height images of eMica nanosheets. Scale bars, 500 nm; *z* scale, −5.0 to 5.0 nm. Insert in (**d**) displays the thickness (~1 nm) of eMica nanosheets measured by AFM. **e** TEM image of eMica nanosheets and relative EDX elemental maps of Si, Al, and O in the eMica nanosheets. Scale bars, 200 nm

Owing to the strong binding force between the highly negative surface charges of the tetrahedral sheets and the interlayer cations, exfoliation of ground mica into high-quality nanosheets is much harder than that of other layered clays^28^, such as MTM. Here, cetyltrimethylammonium bromide (CTAB) was first intercalated into the interlayer of ground mica in deionized water (DIW) at 80 °C after pretreatment. The intercalated ground mica was further exfoliated via ultrasonication treatment in ethanol (Fig. 1a and Supplementary Fig. 1). This method is quite different from previously reported liquid-phase exfoliation of natural ground mica, which uses relatively harsh experimental conditions and deleterious chemical reagents^15^. Small-angle X-ray diffraction (XRD) pattern of the intercalated mica shows that the diffraction peak at ~8.9^o^ disappears, while a new diffraction peak at ~1.9° appears (Fig. 1b). This change indicates that the interlayer distance increases from 1 to 4.77 nm, and the intercalated CTAB takes a paraffin-like bilayer configuration with a tilting angle of 48.9° (See Supplementary Discussion for the details). In contrast, no diffraction peak was found for the eMica nanosheets, confirming that eMica nanosheets do not exist as a layered structure. Successful exfoliation was further confirmed via wide-angle XRD (Supplementary Fig. 2a). The typical morphology of the eMica nanosheets is shown as Fig. 1c, and their thickness was measured to be ~1 nm (Fig. 1d). These experimental observations are entirely different from those for ground mica (Supplementary Fig. 3), providing another strong evidence for successful exfoliation^15^. The theoretical thickness of single layer mica is ~1 nm^28^, so most of the as-prepared eMica nanosheets are mono-layered. Further analysis revealed that the phase (Supplementary Fig. 2a) composition (Fig. 1e, Supplementary Fig. 2b and Supplementary Table 1) and functional groups (Supplementary Fig. 2c) all did not change after exfoliation. In addition, both X-ray photoelectron spectroscopy (XPS) and Fourier transform infrared spectroscopy (FTIR) results can confirm the absence of CTAB on the surface of the obtained eMica nanosheets (Supplementary Fig. 2b, c).

**Fig. 2:**
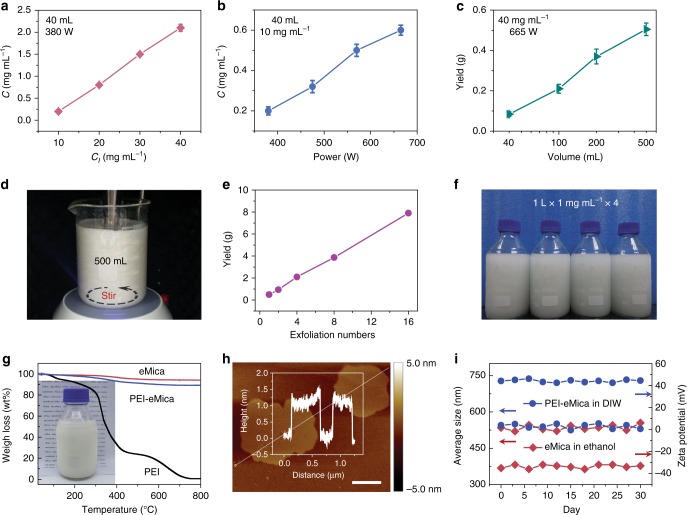
Large-scale exfoliation of ground mica into eMica nanosheets. **a** Concentrations (*C*) of eMica nanosheet dispersions prepared by 30 min ultrasonication treatment (380 W) of 40 mL of intercalated mica dispersions with different initial concentrations (*C*_*I*_). **b** Concentrations of eMica nanosheet dispersions prepared by 30 min ultrasonication treatment of 40 mL of intercalated mica dispersions (10 mg mL^−1^) with different ultrasonication powers. **c** Yields of eMica nanosheets prepared by 30 min ultrasonication treatment (665 W) of different volumes of intercalated mica dispersions (40 mg mL^−1^). **d** Photograph of a 15 mm ultrasonic horn with ultrasonication power of 665 W in 500 mL of intercalated mica dispersion with magnetic stirring. **e** Yields of eMica nanosheets under certain conditions including *C*_*I*_ (40 mg mL^−1^), intercalated mica dispersion volume (500 mL), and ultrasonication power (665 W) as a function of exfoliation number (0.5 h for each exfoliation process). **f** Photograph of the eMica nanosheets dispersed in 4 L ethanol produced within 4 hours with a concentration of eMica nanosheets of ~1 mg mL^−1^. **g** TGA curves of eMica nanosheets, PEI, and PEI-eMica nanosheets. Inset is a digital photograph of the DIW dispersion of PEI-eMica nanosheets with a concentration of 10 mg mL^−1^. **h** AFM height image of PEI-eMica nanosheets. Insert displays that the thickness of PEI-eMica nanosheets is measured to be ~1.2 nm. Scale bar, 200 nm; *z* scale, −5.0 to 5.0 nm. **i** Particle size distributions and zeta potential values of the eMica nanosheets dispersed in ethanol and the PEI-eMica nanosheets dispersed in DIW dispersion measured every three days for one month. All the error bars represent the s.d. of at least five replicate measurements

The scalability and reproducibility of the exfoliation process were further investigated. The yield of the prepared eMica nanosheets depends strongly on the initial content of intercalated mica in the dispersion, the ultrasonication power, and the dispersion volume (Fig. 2a–c). As shown in Fig. 2a, b, the concentrations of the prepared eMica nanosheet dispersions scale linearly with the initial concentration of the intercalated mica and the ultrasonication power. When a larger volume (500 mL) dispersion is treated under higher ultrasonication power, a high production rate of ~1.0 g h^−^^1^ can be easily achieved (Fig. 2c). Large-scale exfoliation process based on a 500 mL of dispersion with an initial intercalated mica concentration of 40 mg mL^−^^1^ under 665 W ultrasonication power is displayed in Fig. 2d. Based on this process, the eMica nanosheets dispersed in 4 L ethanol with concentration of ~1.0 mg mL^−^^1^ were prepared during a period of 4 h and stored in glass bottles (Fig. 2e, f). Moreover, the production rate (~1.0 g h^−1^) of the eMica nanosheets is much higher than that of many other thin nanosheets prepared via previously reported exfoliation processes (Supplementary Table 2). We expect that this exfoliation process can be used to fabricate much greater amount of eMica nanosheets for practical applications when extended from the laboratory scale to the industrial scale.

To enhance the stability of eMica nanosheets in the DIW dispersion, polyethyleneimine (PEI) was used to modify the surface of the eMica nanosheets. The zeta potential of the eMica nanosheets was found to change from negative (approximately −32.23 mV) to positive (approximately 44.20 mV) through PEI modification (Supplementary Table 3). The content of PEI in modified eMica nanosheets was determined to be ~4.8 wt.% by thermal gravimetric analysis (TGA) (Fig. 2g). The thickness changed from ~1 nm (eMica nanosheets) to ~1.2 nm after PEI modification (Fig. 2h). Atomic force microscopy (AFM), transmission electron microscopy (TEM), and dynamic light scattering measurements confirmed no aggregation of the PEI-modified eMica (PEI-eMica) nanosheets (Fig. 2h and Supplementary Fig. 4). Moreover, the stability of the eMica nanosheets in ethanol and PEI-eMica nanosheets in DIW was further evidenced by stable particle size distributions and zeta potential values tested during the course of one month (Fig. 2i).

### Construction of transparent polymeric mica films

**Fig. 3:**
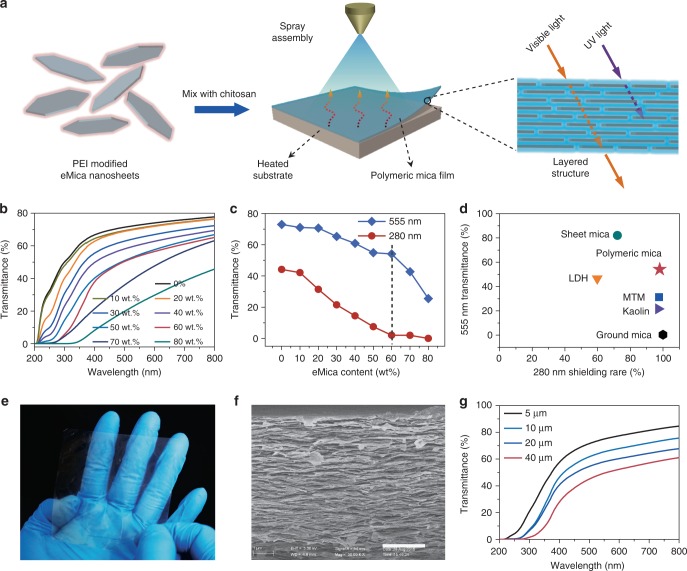
Optical performance of polymeric mica films. **a** Schematic illustration displaying that PEI-eMica nanosheets are mixed with CS solution, which is further assembled into transparent and strong polymeric mica films by a spray-coating method. **b** UV-visible transmittances of ~25 µm thick polymeric mica films with different eMica nanosheet content. **c** Transmittances of polymeric mica films at 555 nm (visible region) and 280 nm (UV region) with different eMica nanosheet content. **d** Comparison of the transmittance at 555 nm and shielding rate at 280 nm of 60 wt.% polymeric mica film, sheet mica, and other as-prepared nacre-like competitors with same thickness (~25 µm). **e** Photograph of a typical 60 wt.% polymeric mica film with a large area (64 cm^2^), which shows prominent transparency. **f** Cross-sectional SEM image of the 60 wt.% polymeric mica film. Scale bar, 2 μm. **g** UV-visible transmittances of 60 wt.% polymeric mica films with varying thicknesses from 5 to 40 µm

The integration of nano-building blocks into macroscopic assemblies is highly desirable for realizing their practical applications^31^. We assembled the PEI-eMica nanosheets with CS into nacre-like polymeric mica films by using a facile spray-coating method (Fig. 3a). A series of polymeric mica films with increasing eMica nanosheet content (from 0 to 80 wt.%) in a CS matrix were prepared (Supplementary Fig. 5a). The polymeric mica films with eMica nanosheet content from 0 to 60 wt.% were transparent. When the eMica nanosheet content reached 70 wt.%, the visible transmittance sharply decreased. UV-visible spectra were measured to quantitatively investigate the transmittance of those polymeric mica films. Spectra in the visible region indicate slow reduction of visible transmittance as the eMica nanosheet content rises to 60 wt.%, but the visible transmittance of the 70 wt.% and 80 wt.% polymeric mica films abruptly drops, probably due to aggregation of eMica nanosheets at high loading content (Fig. 3b). The transmittance in the visible region of polymeric mica film is much higher than that of nacre-like composite films assembled from ground mica, MTM, kaolin, and Mg-Al-LDHs with the same inorganic content and thickness (Supplementary Figs. 5b and 6). This observation is partly attributed to the intrinsic visible transparency of mica itself. In addition, the visible transmittance of 60 wt.% polymeric mica film (38–65%) is comparable to that of synthetic sodium-mica-poly (vinyl alcohol) composite film (45–75%)^27^. Figure 3c reveals that the transmittance of the 60 wt.% polymeric mica film at 555 nm (visible) and 280 nm (UV) exhibits optical selectivity. Namely, the film possesses both excellent visible transparency and nearly complete UV-shielding (Supplementary Fig. 5c). This unique optical performance has not been observed in previously reported nacre-like films, and is superior to that of other as-prepared nacre-like competitors and comparable to natural sheet mica (Fig. 3d). This selectivity is partly ascribed to the layered crystalline structure^25^ and to the polarization and interlayer light interference of mica^14,32^. The 60 wt.% polymeric mica film can be fabricated on many different substrates to protect them from UV damage due to its UV-shielding property (Supplementary Fig. 7). Furthermore, as shown in Fig. 3e, a large transparent and flexible 60 wt.% polymeric mica film (8 cm × 8 cm) can be obtained with straightforward processing.

Apart from the unique visible transparency of mica itself, the superior visible transmittance of these polymeric mica films is predominantly attributed to their ordered layered microstructure^33^. Scanning electron microscopy (SEM) observations of their fracture surfaces (Fig. 3f and Supplementary Fig. 5d–f) showed clearly layered “brick-and-mortar” structures in these polymeric mica films, while no layered structure can be observed in pure CS film (Supplementary Fig. 8a). A skin layer typically formed on the surface of films fabricated through evaporation^27^. In contrast, no structural distortion was observed in spray induced polymeric mica films (Fig. 3f). When eMica nanosheets were replaced by ground mica in a 60 wt.% ground mica composite film, inferior visible transmittance and layered microstructure were observed (Supplementary Fig. 8b), and are mainly attributed to the large size and low aspect ratio of ground mica. The eMica nanosheet content in these polymeric mica films was further confirmed by TGA (Supplementary Fig. 9a). As shown in Supplementary Figs. 8c and 9b, the enhanced diffraction intensity in the XRD patterns of the polymeric mica films is similar to that of the natural ground mica, possibly due to the reconstructed layered structure of eMica nanosheets in these films. Small-angle XRD patterns further reveal the layered microstructure of these polymeric mica films (Supplementary Fig. 9c). The homogeneous distribution of the various constituents, obtained by SEM energy-dispersive X-ray elemental spectrum and mapping of the fracture surface of the 60 wt.% polymeric mica film, confirms its uniform structure (Supplementary Fig. 9d–h). Moreover, 60 wt.% polymeric mica films with different thicknesses were easily produced by controlling the volume of dispersions sprayed (Supplementary Fig. 10). Polymeric mica films with smaller thicknesses were found to display superior visible transmittance (Fig. 3g).

### Mechanical properties of polymeric mica films

**Fig. 4:**
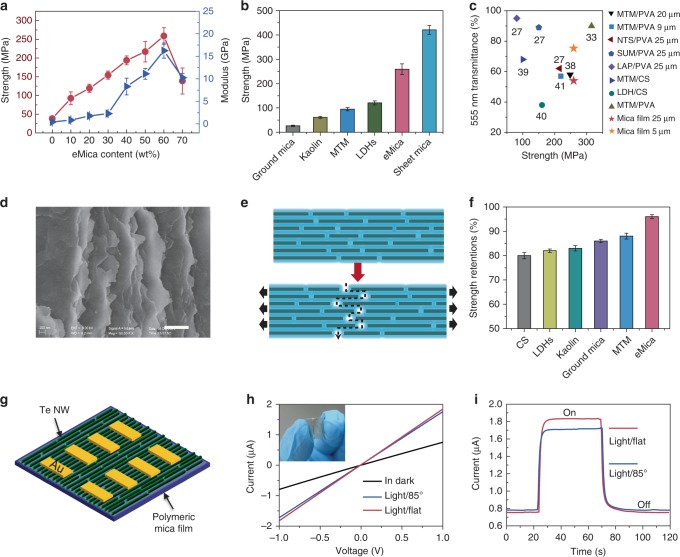
Mechanical performance investigation and flexible and transparent device fabrication. **a** Influence of eMica nanosheet content on the tensile strength and Young’s modulus of these films. **b** Comparison of the tensile strength of 60 wt.% polymeric mica film, sheet mica, and other as-prepared nacre-like competitors with same thickness (~25 µm). **c** Comparison of strength and transmittance at 555 nm of the 60 wt.% polymeric mica film and other nanoclay-based biomimetic composite films^27,33,^^38–41^. **d** Fracture morphology of the 60 wt.% polymeric mica film after tensile testing. Scale bar, 800 nm. **e** Proposed synergistic mechanical enhancement mechanism of the 60 wt.% polymeric mica film. **f** Retention of the tensile strength of 60 wt.% polymeric mica and other as-prepared nacre-like competitors after UV irradiation for 144 h. **g** Schematic illustration of the device with 60 wt.% polymeric mica film as its substrate. **h** Typical I–V curves of the device measured in the dark and upon light illumination (450 nm, 180 mW cm^−2^, flat and bent). Inset shows a photograph of a bent device. **i** Time response spectrum of the device to pulsed light at V = 1 V. All the error bars represent the s.d. of at least five replicate measurements

As previously reported, natural sheet mica possesses particularly favorable crystallinity, a perfect layered structure (Supplementary Fig. 11), and mechanical performance superior to that of MTM^34^. Its tensile strength was found to be as high as ~420 MPa, which is comparable to that of structural steel (ASTM A36 steel, 400 MPa). However, natural sheet mica is relatively brittle, which is easily broken after only a few bending cycles due to interlayered cleavage. In contrast, polymeric films generally exhibit high flexibility but low mechanical strength, so it is expected to be of great significance to integrate the advantages of eMica nanosheets into flexible and strong polymeric mica films. Relative to pure CS film, which has a low tensile strength (38.4 MPa) and Young’s modulus (0.45 GPa) (Fig. 4a and Supplementary Fig. 12a), the as-prepared polymeric mica films possess enhanced tensile strength and Young’s modulus with increasing eMica nanosheet content. The optimal mechanical strength and Young’s modulus (approximately 259 MPa and 16.2 GPa, respectively) are achieved when the eMica nanosheet content reached 60 wt.% (Fig. 4a). As shown in Fig. 4b, the tensile strength of 60 wt.% polymeric mica film is higher than that of other as-prepared nacre-like composite films and reaches up to 62% of that of natural sheet mica. The tensile strength of composite films assembled from CS modified eMica nanosheets is significantly lower than that of polymeric mica films assembled from PEI-eMica with the same inorganic content (Supplementary Fig. 13a). This finding is mainly attributed to the denser amino groups in the molecular chains of PEI relative to CS, which leads to stronger electrostatic interaction between the PEI and the eMica nanosheets and dense hydrogen bonds between the PEI-eMica nanosheets and the CS matrix (Supplementary Fig. 13b-e and Supplementary Table 3). Compared with previously reported biomimetic films, the 60 wt.% polymeric mica film shows an excellent combination of both high strength and visible (555 nm) transmittance (Fig. 4c and Supplementary Table 4). The 60 wt.% polymeric mica film exhibits not only unique optical selectivity but also maximal enhancement of mechanical properties. We attribute the superior mechanical enhancement of the 60 wt.% polymeric mica film (relative to the nacre-like films assembled from other nanoclay and ground mica) to the intrinsic mechanical properties and large aspect ratio of the eMica nanosheets^27^. The fracture surface of the 60 wt.% polymeric mica film shows abundant pull-out of eMica nanosheets after tensile failure and a typical trapezoidal structure and crack deflection are observed in the fracture surface (Fig. 4d, e and Supplementary Fig. 12b). These characteristics are considered to be the major structural enhancement mechanisms for polymeric mica film^23,31^. Moreover, the stable mechanical strength of the 60 wt.% polymeric mica film after a cyclic bending test certifies its superior flexibility and fatigue resistance (Supplementary Fig. 14).

The unique UV-shielding performance of the polymeric mica film endows it with strong resistance to UV ageing. When the 60 wt.% polymeric mica film was exposed to UV irradiation, almost no change in tensile strength (~4% degeneration) was detected (Fig. 4f). In contrast, pure CS film and other as-prepared nacre-like films all showed larger degradation of tensile strength (from 10 to 20%). In addition, SEM observations revealed that many tiny cracks appeared on the surface and cross section of CS film after 144 h UV irradiation (Supplementary Fig. 15), while the 60 wt.% polymeric mica maintained satisfactory microstructure morphology (Supplementary Fig. 16).

### Serving as a substrate for flexible photoelectric device

Natural mica is an excellent insulating material^16^, and the electronic resistance of the 60 wt.% polymeric mica film was measured to be much higher than that of pure CS film (Supplementary Fig. 17a). Moreover, the tensile strength of our polymeric eMica film was found to be three times higher than that of polyethylene terephthalate film (60 MPa) (Supplementary Fig. 17b). These attractive mechanical and optical properties together with excellent electronic property make the 60 wt.% polymeric mica film a promising substrate for flexible and transparent devices. As a potential application, a typical flexible photoelectric device illustrated in Fig. 4g was fabricated based on this substrate. A tellurium nanowire (Te NW) monolayer was fabricated on the surface of the flexible 60 wt.% polymeric mica film by the Langmuir-Blodgett (LB) procedure, in accordance with our previous report^35^, and then gold electrodes were deposited on the substrate by e-beam evaporation. Figure 4h, i, shows typical current (I)-voltage (V) curves and time response spectra of the as-synthesized device. Note that conductivity of the flat Te NW film was tested to be ~0.24 S cm^−1^ in the dark while increased to ~0.6 S cm^−1^ when the flat Te NW film was exposed to visible blue light (450 nm, power density 180 mW cm^−^^2^), showing photoconduction behavior comparable to the device counterparts on silicon oxide/silicon substrates^35^. Furthermore, the device maintained well performance over a wide range of bending angles (0–85°), indicating that the as-synthesized polymeric mica film can work well as a flexible substrate for electronic devices.

## Discussion

In summary, large-scale exfoliation of ground mica into high-quality mono- or few-layered eMica nanosheets was successfully achieved with a production rate of ~1.0 g h^−1^. The eMica nanosheets were further assembled into biomimetic polymeric mica films with a nacre-like layered structure. Owing to the intrinsic properties and the high level of ordering of the eMica nanosheets in the film, the obtained polymeric mica film with optimum eMica nanosheet content (60 wt.%) exhibits excellent mechanical properties, high electric insulation, and unique UV-visible selectivity. In particular, its unique UV-shielding performance endows it with impressive resistance to UV ageing. Its overall performance is superior to that of natural sheet mica and other kinds of clay-based biomimetic films, making the polymeric mica film a suitable candidate to serve as novel substrate for flexible and transparent electronic devices, such as photodetector presented in this work. Our study offers a scalable method for the fabrication of high-quality eMica nanosheets, and opens an avenue for strong application potentials of the polymeric mica film, especially in the field of electronic and electrical industries.

## Methods

### Materials

Natural ground mica was obtained from Anhui Gerui Co. Ltd. (Chuzhou, Anhui, China). CS and kaolin were purchased from Aladdin Chemical Reagent Co. PEI (Mw = 25,000) was purchased from Sigma-Aldrich. Glacial acetic acid, ethanol, methanol, dimethyl sulfoxide (DMSO), N, N-dimethylformamide (DMF), nitric acid (HNO_3_), CTAB, sodium hydroxide (NaOH), sodium chloride (NaCl), aluminum chloride, and magnesium chloride were purchased from Sinopharm Chemical Reagent Co. MTM clays were obtained from Zhejiang Hongfeng Clay Co. Ltd. (Huzhou, Zhejiang, China).

### Exfoliation of ground mica and MTM and synthesis of LDHs

For exfoliation of ground mica, it was first intercalated by CTAB in a DIW solution at 80 °C^12^, and the product was named as CTAB-mica. In a typical procedure, 10 g of ground mica was heated by furnace at 800 °C for 1 h under air, then 3 g of the resultant mica powder was dispersed into 100 mL of DIW containing 5.0 mol L^−1^ HNO_3_ at 95 °C, stirred for 5 h, filtered and washed with thermal DIW (~80 °C) for several times to be neutral, and then dried at 80 °C. Subsequently, 3 g of the obtained powder was dispersed into 100 mL of saturated NaCl solution at 95 °C, stirred for 3 h, filtered and washed with thermal DIW (~80 °C) for several times to remove redundant NaCl, and then dried at 80 °C. Then, 1.5 g of the resulting powder and 4.6 g of CTAB were dispersed into 150 mL of DIW at 80 °C, stirred for 24 h, filtered and washed with thermal DIW (~80 °C) for several times to remove redundant CTAB, and then dried at 80 °C. After intercalation process, a certain amount of CTAB-mica was then dispersed into ethanol (40–500 mL), followed by ultrasonication at various powers (380–665 W) for 30 min. The supernatant containing eMica nanosheets was obtained by centrifuging the above dispersion at 940 × g for 10 min to remove the unexfoliated CTAB-mica. Na-MTM was exfoliated by using mechanical stirring in DIW for 1 week^10^. Mg-Al-LDHs were synthesized by using hydrothermal treatment at a temperature of 100 °C for 10 h^36^.

### Preparation of biomimetic polymeric mica films and other nacre-like films

The obtained ethanol dispersion of eMica nanosheets was first centrifuged (10,380 × g, 10 min) to collect the eMica nanosheets. The collected precipitate was washed with DIW for several times to remove the ethanol, and then dispersed into DIW. Subsequently, the eMica nanosheets were modified with PEI using a modified method described in an earlier work^37^. In a typical process, 100 mg of PEI and 292 mg of NaCl were dissolved in 500 mL of a dispersion of eMica nanosheets in DIW (10 mg mL^−1^), followed by stirring for 1 h. The resultant PEI-eMica was centrifuged, washed with DIW for several times, and then dispersed into appropriate amount of DIW again. The desired amount of CS solution (2 wt.%) was gradually added into DIW dispersion of PEI-eMica under constant stirring for ~0.5 h, followed by using vacuum pumping to remove the air bubbles. After that, this homogeneous eMica-CS dispersion was poured into a spray lance and then sprayed onto glass substrates placed on a heating stage (90 °C) for preparation of films with a certain thickness. Finally, freestanding polymeric mica films were peeled off from the substrates by using a razor blade. The MTM-CS, kaolin-CS, LDHs-CS, and ground mica-CS nacre-like films were prepared by the same method described above.

### Device fabrication and performance analysis

A monolayer Te NW film formed by the LB technique was transferred to the surface of the as-synthesized 60 wt.% polymeric mica film. E-beam evaporation was used to define the Au (50 nm) electrodes onto the Te NW film through a shadow mask, vertical to the axial direction of the Te NW with a spacing of 8 mm. Electrical characterization was performed on a semiconductor characterization system (Keithley 4200-SCS) at room temperature using a two-probe measurement.

### Sample characterizations

AFM images were obtained by Dimension Icon. TEM images were acquired using a Hitachi H-7650 and a JEM-2100F with an acceleration voltage of 100 kV. SEM images were collected by field emission SEM (Zeiss Supra 40 and Zeiss Merlin compact) at an acceleration voltage of 5 kV. XRD experiments were performed on a Philips X’Pert PRO SUPER X-ray diffractometer equipped with a Cu Kα radiation source. The zeta potential values and size distributions of different materials were determined using a Nano Particle Analyzer (Malvern Nano-2590). The UV-visible spectra of different films were obtained by a SHIMADZU UV-2600. The binding energies of the elements in the materials were measured with XPS using ESCALAB 250Xi. FTIR spectra were obtained using a Thermo Nicolet 6700. TGA experiments were carried out with a STA449F3 thermal analyzer with a heating rate of 10 °C min^−1^ under air. CTAB-mica dispersion was performed on an Ultrasonic Cell Disruption System (JY92-IIDN). Tensile properties were measured using an Instron 5565 A equipped with a 500 N load cell at a load speed of 10 mm min^-1^ and at room temperature with ~50% relative humidity (RH). At least 5 specimens with the size of approximately 30 mm × 3 mm and a thickness of ~25 μm were measured for each group. Fatigue resistance tests were conducted by bending the samples for 10, 100, and 1000 cycles, respectively, with a bending radius of 2.0 mm and bending frequency of 0.5 Hz, and then measuring their tensile strength. UV radiation experiments of pure CS, 60 wt.% polymeric mica film, and other nacre-like films were performed on an accelerated ageing tester (Y/UVB-313). At least 5 films (about 4 cm × 3 cm in size and 25 μm in thickness) for each group were exposed to UV radiation for different times at 30 °C and 50% RH (ASTM G155), and then collected for the following mechanical tests and SEM observations.

### Data availability

The data that support the findings of this study are available on request from the corresponding authors (S.-H.Y. or Y.L).

## Electronic supplementary material

Supplementary Information
