# Supplementary material for: Transforming ground mica into high-performance biomimetic polymeric mica film
Source: Nat Commun. 2018 Jul 30;9:2974. doi: 10.1038/s41467-018-05355-6 (PMC6065438; doi:10.1038/s41467-018-05355-6)
Supplement: Supplementary file 1 — Supplementary Information [file 41467_2018_5355_MOESM1_ESM.pdf]

Supplementary Information

Transforming Ground Mica into High-Performance Biomimetic Polymeric Mica Film

Pan et al.

Supplementary Discussion

Analysis the assembly manner of the CTAB in mica interlayer

The tilting angle θ of the CTAB chains in the interlayer space could be calculated by equation 1[1]:

$$\sin\theta = \frac{d\text{-}d_0}{2L}, \quad (1)$$

where d and d_0 were the d_{002} values of mica after and before intercalation, respectively, and L was the length of the CTAB chain. For CTAB, the chain length was 2.5 nm. Since the d_{002} value of mica at the actual intercalation condition was 4.77 nm and the initial d_{002} value of mica is 1 nm, it could be speculated that the CTAB in the interlayer space adopted a paraffin-type bilayer configuration and the tilting angle θ with respect to the layer plane was calculated to be 48.9°.

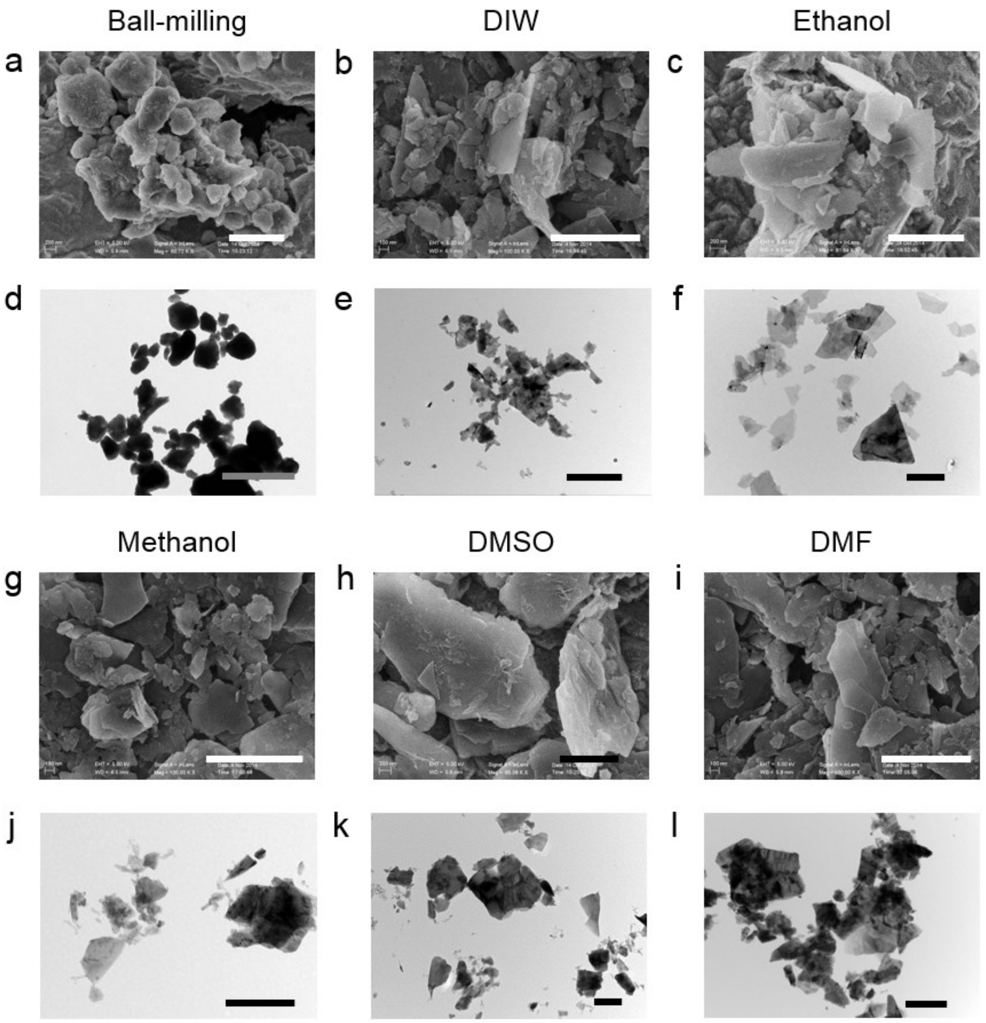

Supplementary Figure 1 | Exfoliation of intercalated mica using different methods and solvents. SEM and TEM images of the products from intercalated mica by ball-milling for 24 h (**a**, **d**), and sonication for one week with concentration of 1 wt.% in DIW (**b**, **e**), ethanol (**c**, **f**), methanol (**g**, **j**), DMSO (**h**, **k**), and DMF (**i**, **l**), respectively. Sonication treatment in ethanol was favor to exfoliate the intercalated mica. *Scale bars*, 1 μm.

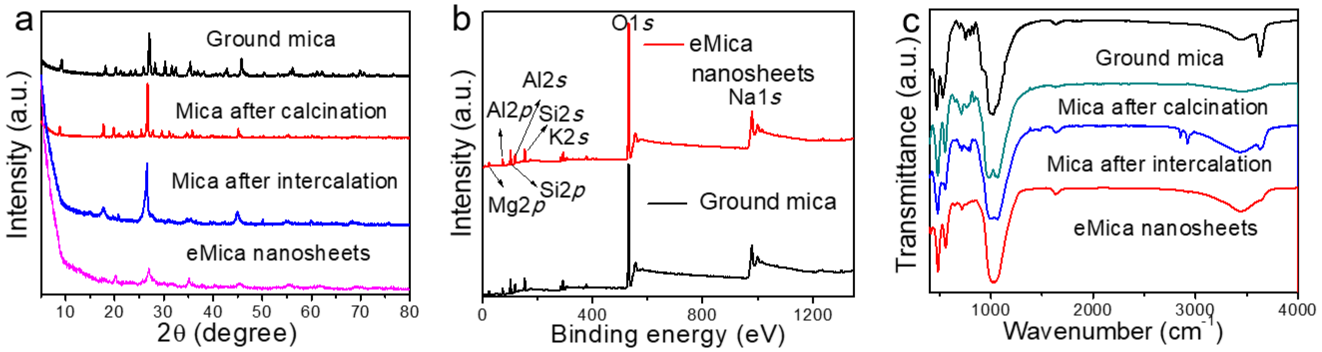

Supplementary Figure 2 | Exfoliation of ground mica into eMica nanosheets. a, XRD patterns of ground mica before and after calcination, intercalation, and exfoliation. After exfoliation, eMica nanosheets exhibit weaker intensity of the diffraction peaks than ground mica, which demonstrates that the layered structure of ground mica collapses. **b**, XPS spectra of ground mica and eMica nanosheets display that the elemental composition of mica does not change before and after exfoliation, and no C element from CTAB can be detected from eMica nanosheets. **c**, FTIR spectra of ground mica before and after calcination, intercalation, and exfoliation, confirming that no functional groups transformation occurs. The peaks at 2918 and 2849 cm^{-1} in the spectrum of intercalated mica correspond to the CH_2 asymmetric and symmetric stretching vibration from CTAB, but the spectrum of eMica nanosheets does not show those peaks[1].

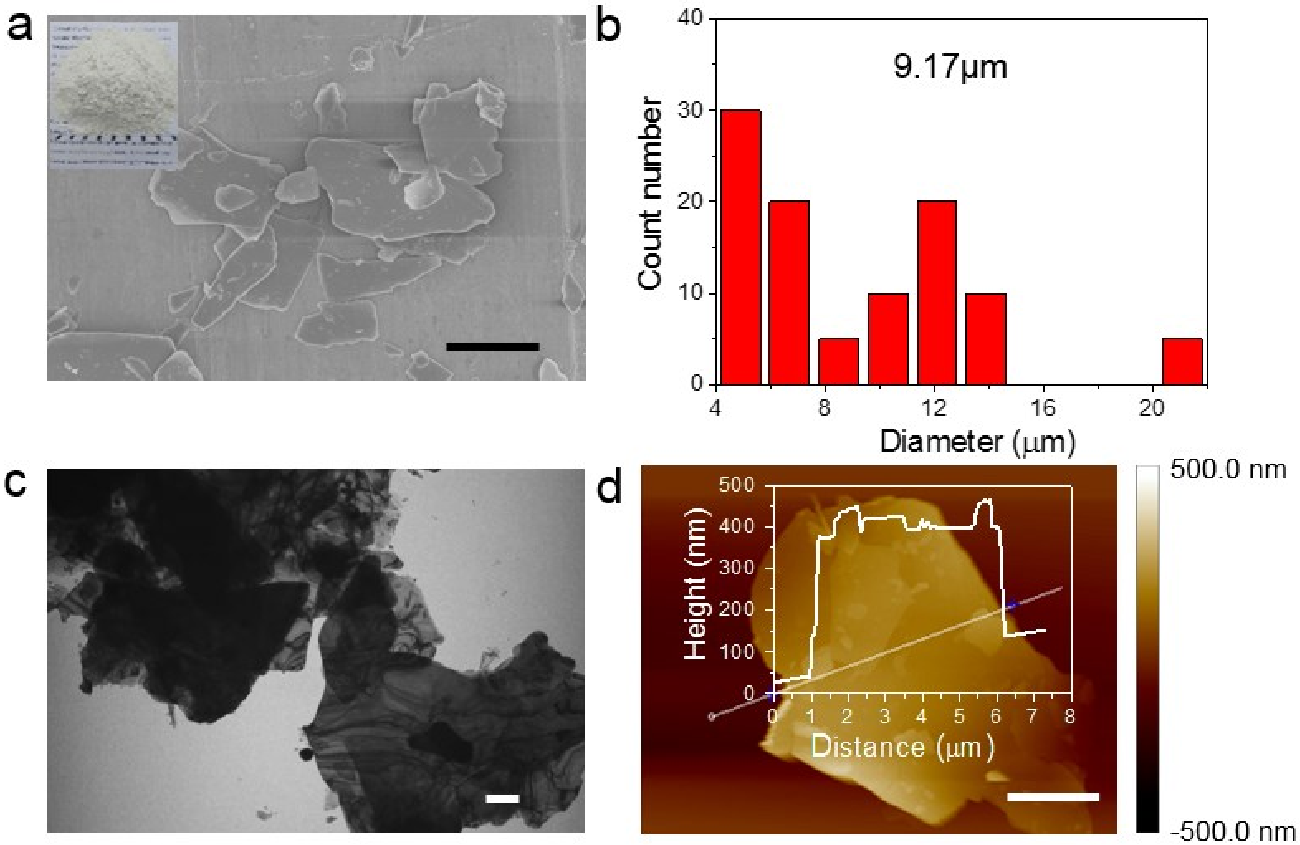

Supplementary Figure 3 │Characterization of ground mica. **a**, SEM image of ground mica. Inset is a photograph of ground mica. *Scale bar*, 10 µm. **b**, Statistical distribution of the lateral sizes of ground mica. The size distribution of ground mica particles was measured by Nano Measurement Software from SEM image, where at least 20 measurements of the ground mica particles were taken. **c**, **d**, TEM and AFM height images of ground mica. Insert in (**d**) shows that its thickness is measured to be about 400 nm by AFM. *Scale bars*, 2 µm; z scale, -500.0 - 500.0 nm.

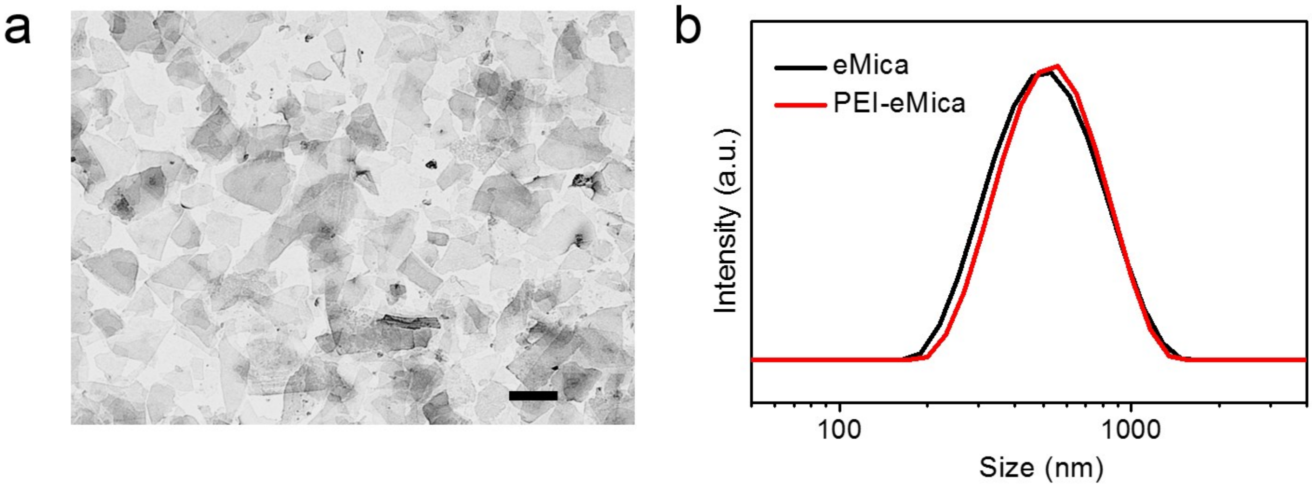

Supplementary Figure 4 │ Characterization of PEI-eMica. **a**, TEM image of PEI-eMica nanosheets. *Scale bar*, 500 nm. **b**, Particle size distributions of PEI-eMica and eMica measured by DLS, confirming almost no change of the particle size after PEI modification.

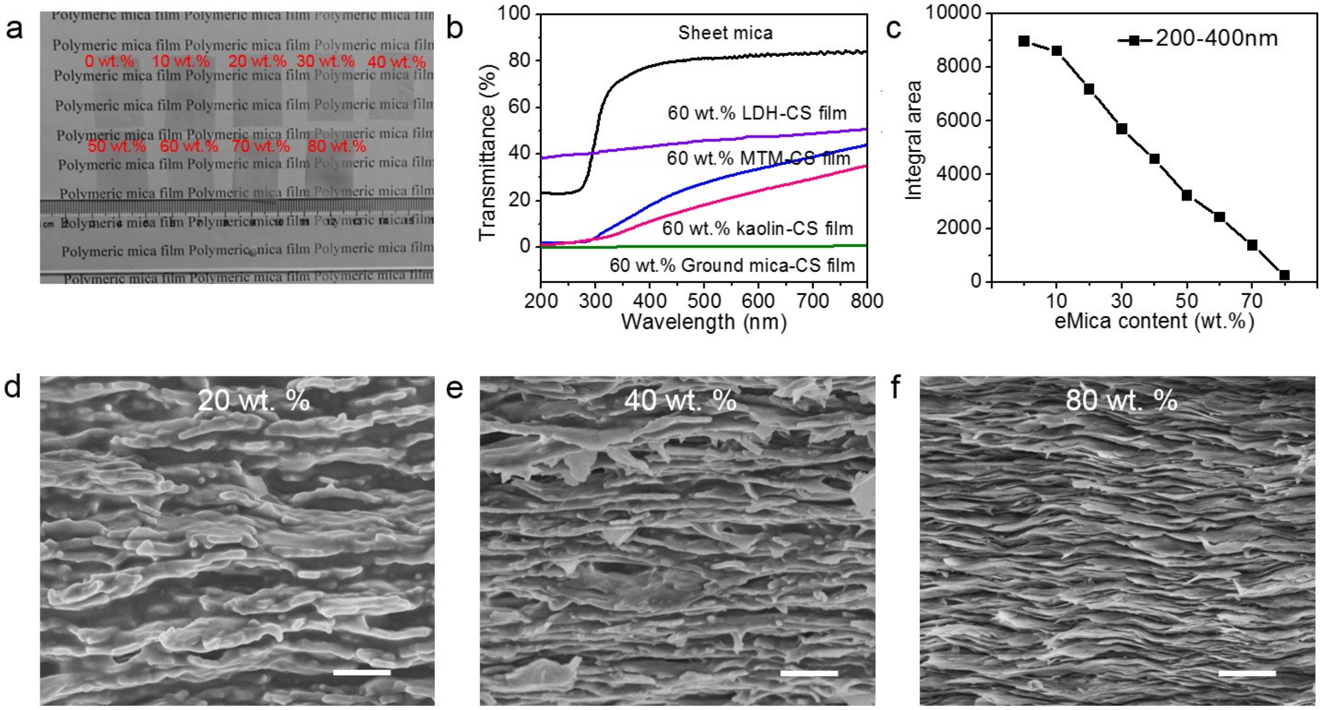

Supplementary Figure 5│Optical and structural characterization of a series of polymeric mica films. **a**, Photographs of a series of polymeric mica films. **b**, UV-visible transmittance spectra of 60 wt.% polymeric mica film, sheet mica, and other as-prepared nacre-like composite films with same thickness of ~25 μm. **c**, Integral areas of a series of polymeric mica films with different eMica nanosheet content in UV region (200-400 nm). The transmittance of these polymeric mica films in UV region was distinctly dropped with increasing eMica nanosheet content. **d-f**, Cross-sectional SEM images of polymeric mica films with 20 wt.%, 40 wt.%, and 60 wt.% of eMica nanosheet content. *Scale bars*, 1 μm.

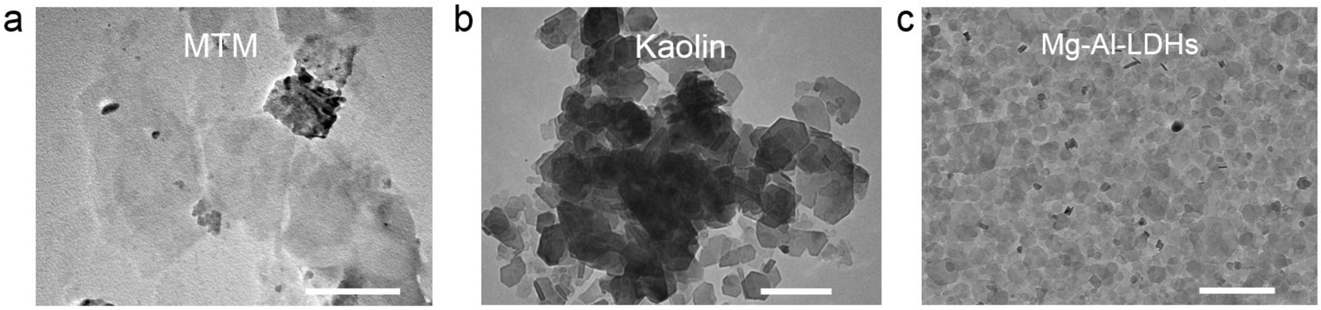

Supplementary Figure 6 | Characterization of nanoclays. a-c, TEM images of MTM, kaolin, and Mg-Al-LDHs used for assembling several other kinds of nacre-like composite films. *Scale bars*, 500 nm.

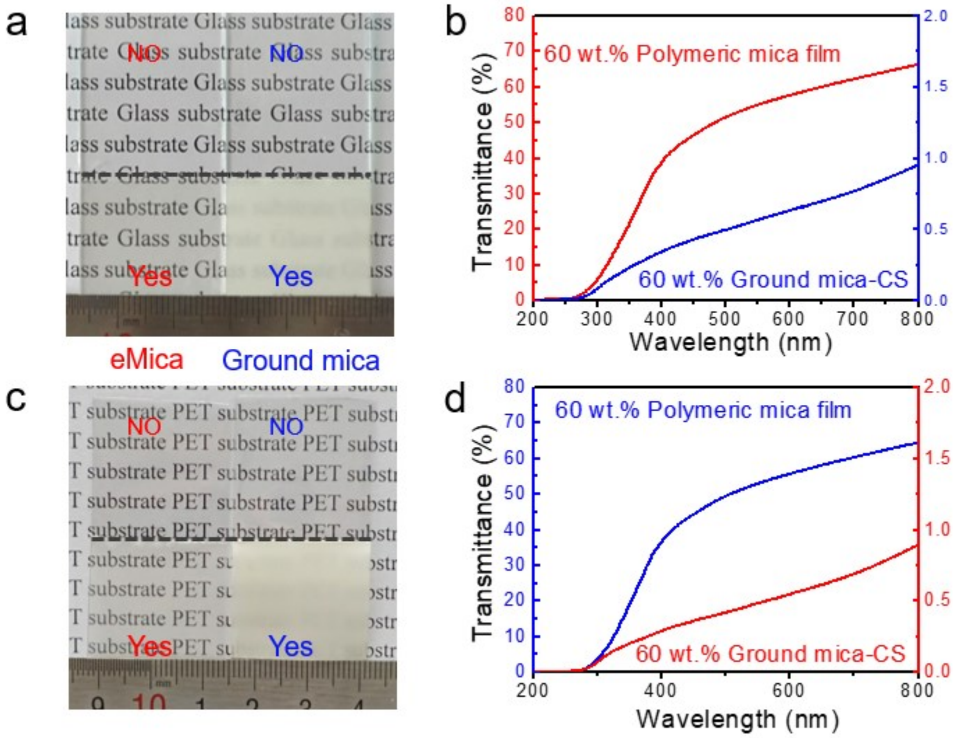

Supplementary Figure 7│Optical characterization of 60 wt.% polymeric mica film prepared on different substrates. **a**, **b**, Photograph (**a**) and UV-visible transmittance spectra (**b**) of 60 wt.% polymeric mica and ground mica-CS composite films on glass substrates. **c**, **d**, Photograph (**c**) and UV-visible transmittance spectra (**d**) of 60 wt.% polymeric mica and ground mica-CS composite films on PET substrates.

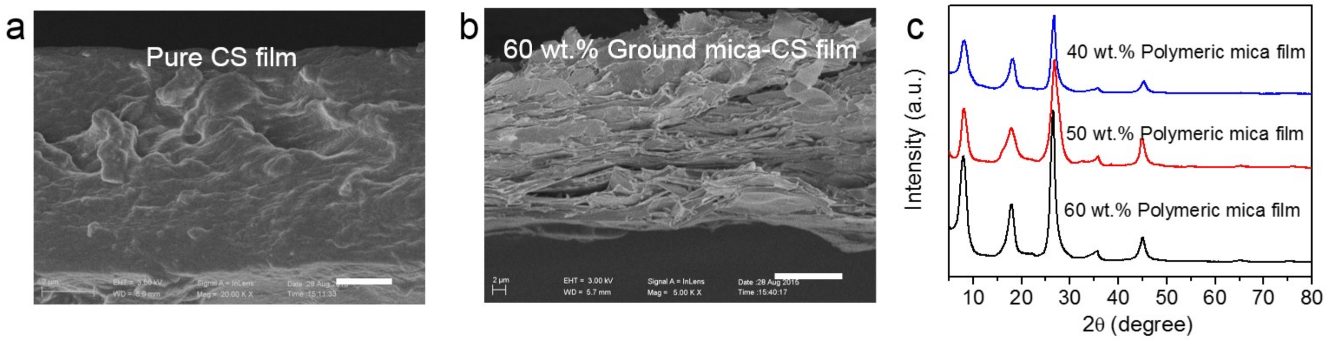

Supplementary Figure 8 | Structural comparison and XRD characterization of different films. a, **b**, The cross-sectional SEM images of CS film and 60 wt.% ground mica-CS film. *Scale bars*, 2 μm and 10 μm, respectively. **c**, XRD patterns of these polymeric mica films with different eMica nanosheet content.

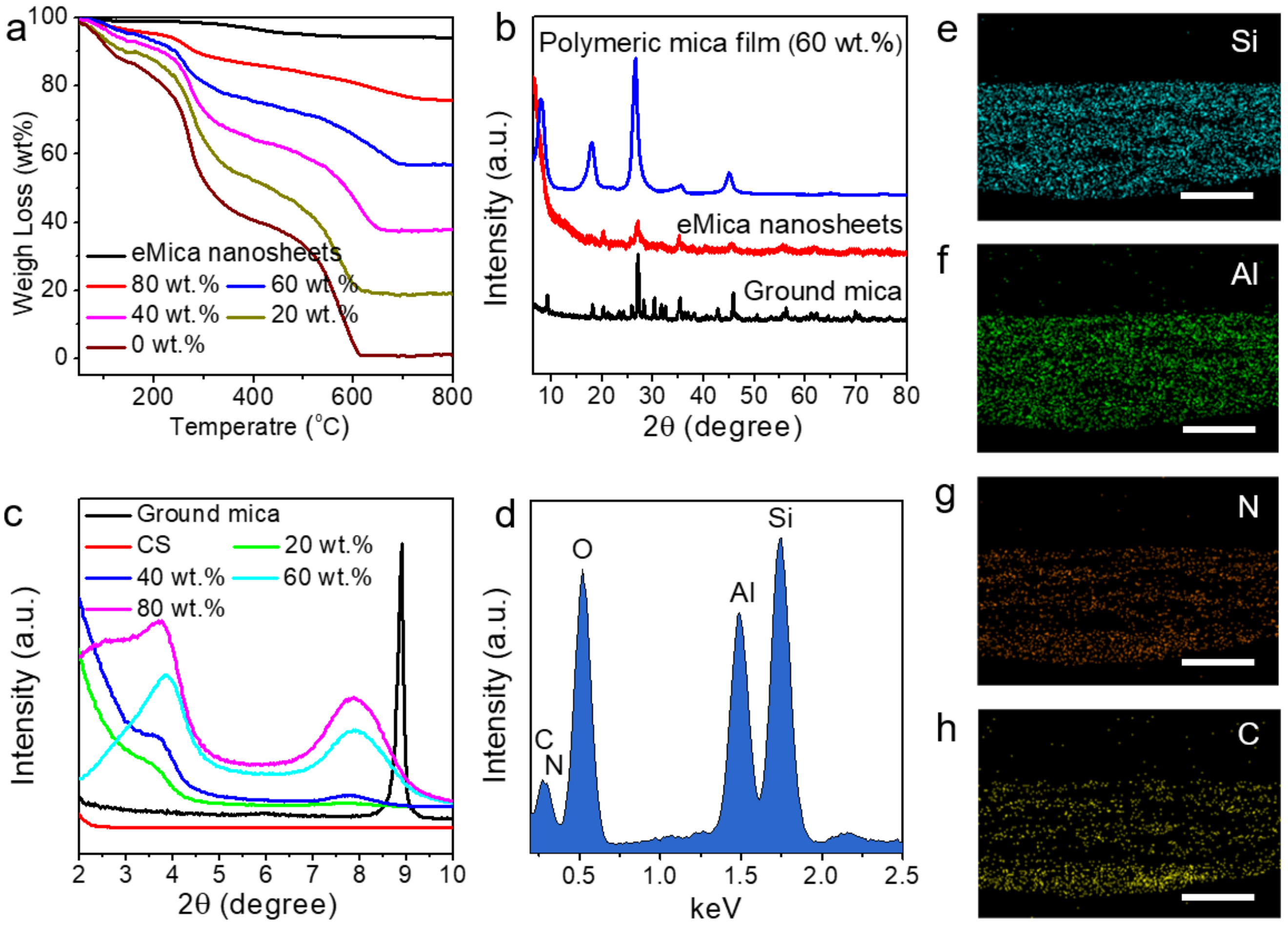

Supplementary Figure 9│Structural characterization of polymeric mica films. a, TGA curves of eMica nanosheets and a series of polymeric mica films. **b**, XRD patterns of ground mica, eMica nanosheets, and 60 wt.% polymeric mica film. **c**, Small-angle XRD patterns of ground mica, CS film, and a series of polymeric mica films. Regarding to 20-40 wt.% polymeric mica films, the presence of diffraction peak at 3.8° indicates the *d* spacing of 2.2 nm, while the new diffraction peak at 7.8° for 60-80 wt.% polymeric mica films indicates the *d* spacing of 1.1 nm. **d-h**, The elemental distribution of a 60 wt.% polymeric mica film is observed in the SEM-EDX elemental spectrum and maps including the presence of Si (**e**), Al (**f**), N (**g**) and C (**h**). The distributions of carbon and nitrogen from chitosan molecules are in perfect correspondence with that of aluminum and silicon from mica. *Scale bars*, 20 μm.

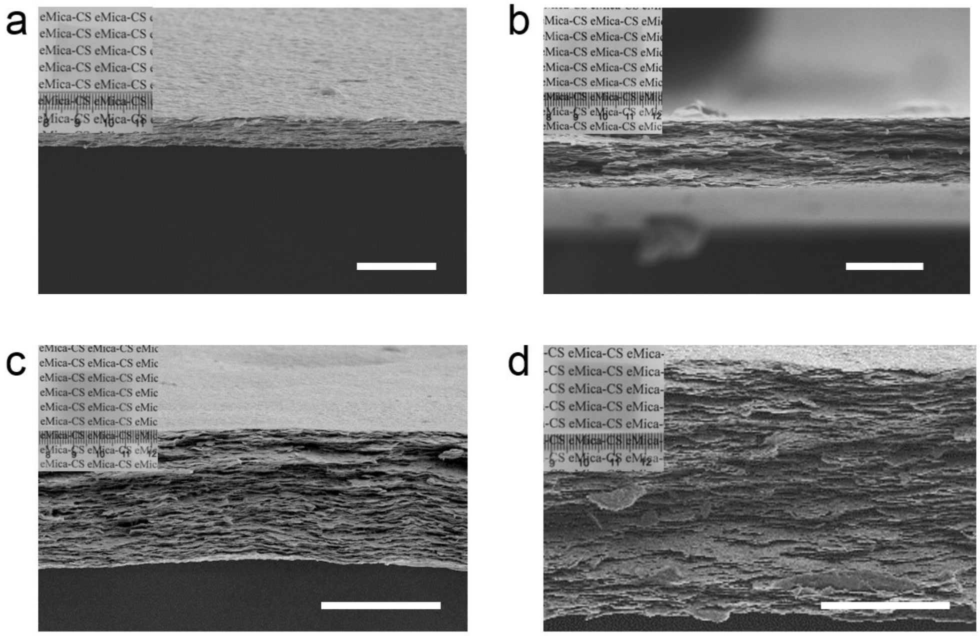

Supplementary Figure 10 | Polymeric mica films with controllable thickness. a-d, Cross-sectional SEM images of 60 wt.% polymeric mica films with varying thicknesses from 5 to 40 μm. Inserts are corresponding to photographs. *Scale bars*, 10 μm.

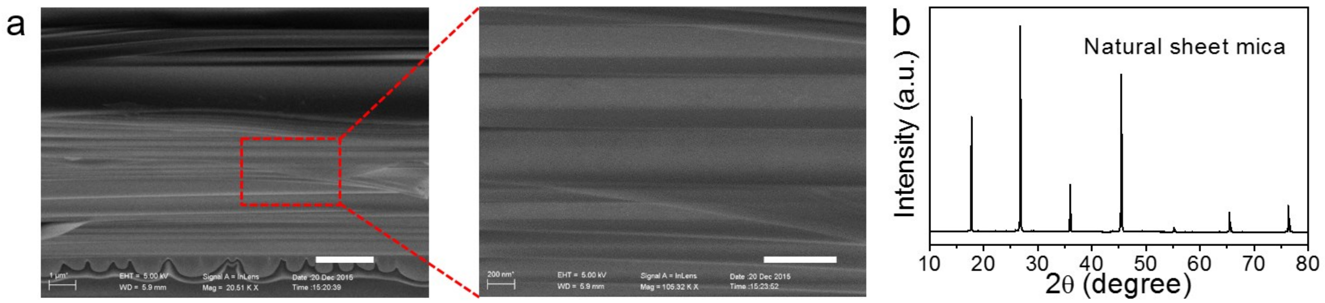

Supplementary Figure 11 │ Characterization of natural sheet mica. a, Cross-sectional SEM images of natural sheet mica. *Scale bars*, 2 μm and 500 nm, respectively. **b**, XRD pattern of natural sheet mica.

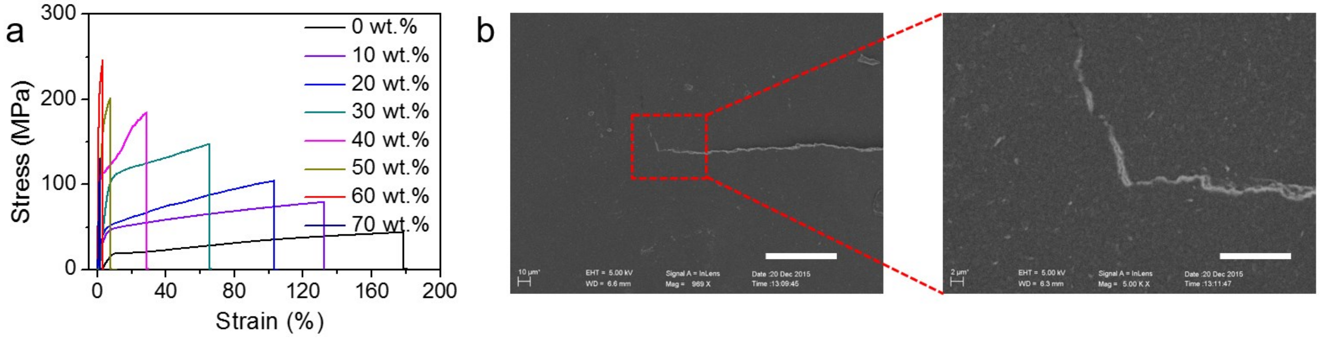

Supplementary Figure 12 │ Mechanical characterization of polymeric mica films. a, Representative stress-strain curves of polymeric mica films with different eMica nanosheet content. **b**, Fracture morphology of 60 wt.% polymeric mica film displays long-distance crack deflection. *Scale bars*, 50 μm and 10 μm, respectively.

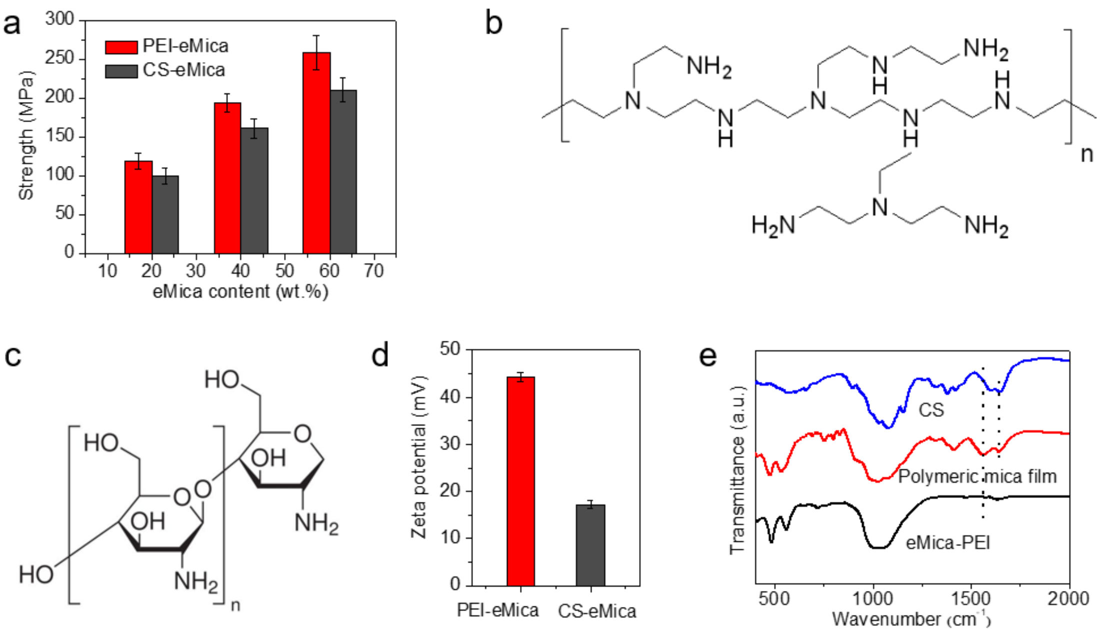

Supplementary Figure 13 | Interface characterization of polymeric mica film. **a**, Comparison of the tensile strength of composite films assembled from eMica nanosheets modified with PEI and CS, respectively. **b**, **c**, Molecular structure of PEI (**b**) and CS (**c**). **d**, Zeta potential values of DIW dispersions of PEI-eMica and CS-eMica. **e**, FTIR spectra of PEI-eMica nanosheets, CS, and 60 wt.% polymeric mica film. The peak at 1656 and 1596 cm^{-1} in the spectrum of CS are characteristics of the C=O stretching vibration of -NHCO- and the N-H bending of $-NH_2$, respectively[2]. Characteristic absorbance band at 1575 cm^{-1} in the spectrum of PEI corresponds to the N-H bending of $-NH_2$[3]. Notably, in the spectrum of polymeric mica film, both the peaks of the C=O stretching vibration of -NHCO- and the N-H bending of $-NH_2$ shift to lower wavenumbers. These results revealed dense hydrogen bonds between CS and PEI-eMica[2]. All the error bars represent the s.d. of at least five replicate measurements.

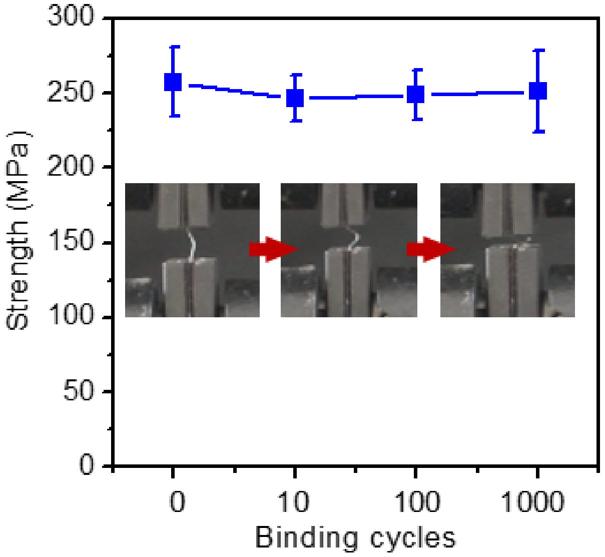

Supplementary Figure 14 | Tensile strength of the 60 wt.% polymeric mica film after 10, 100, and 1000 cycles of bending tests with bending radius of 2.0 mm, respectively. The inset shows the bending process of a 60 wt.% polymeric mica film. All the error bars represent the s.d. of at least five replicate measurements.

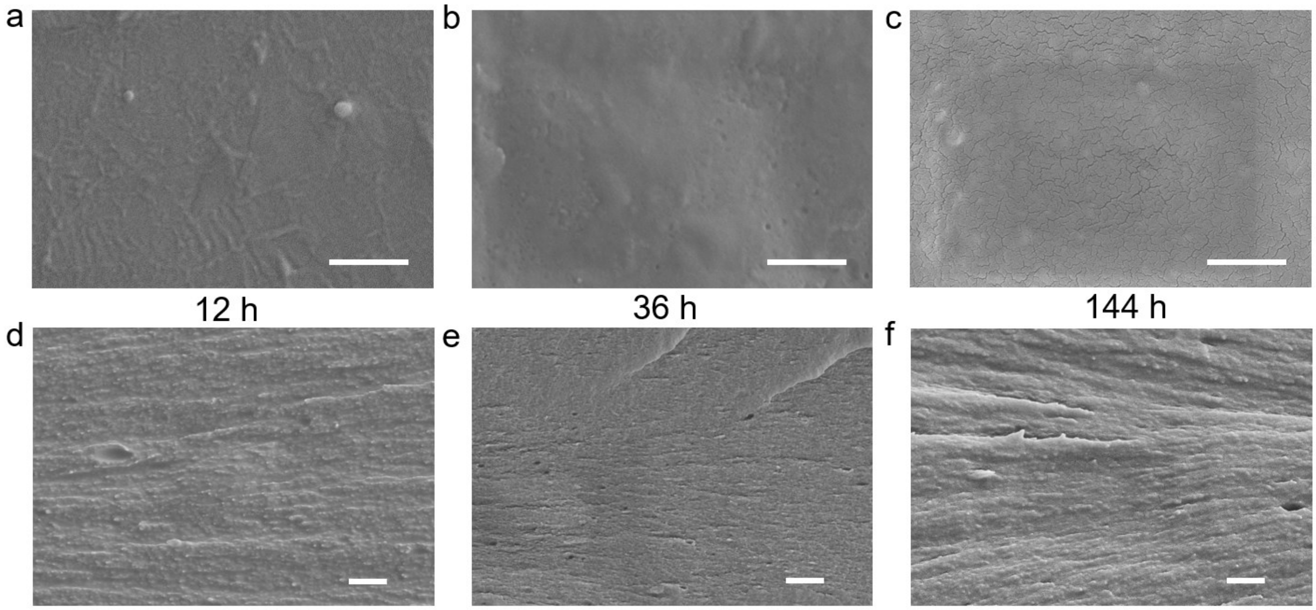

Supplementary Figure 15│Microstructure of CS film after UV irradiation. a-f, SEM images of the surfaces (**a-c**) and cross sections (**d-f**) of pure CS film after 12 h, 36 h, and 144 h UV irradiation, respectively. *Scale bars*, 1 µm.

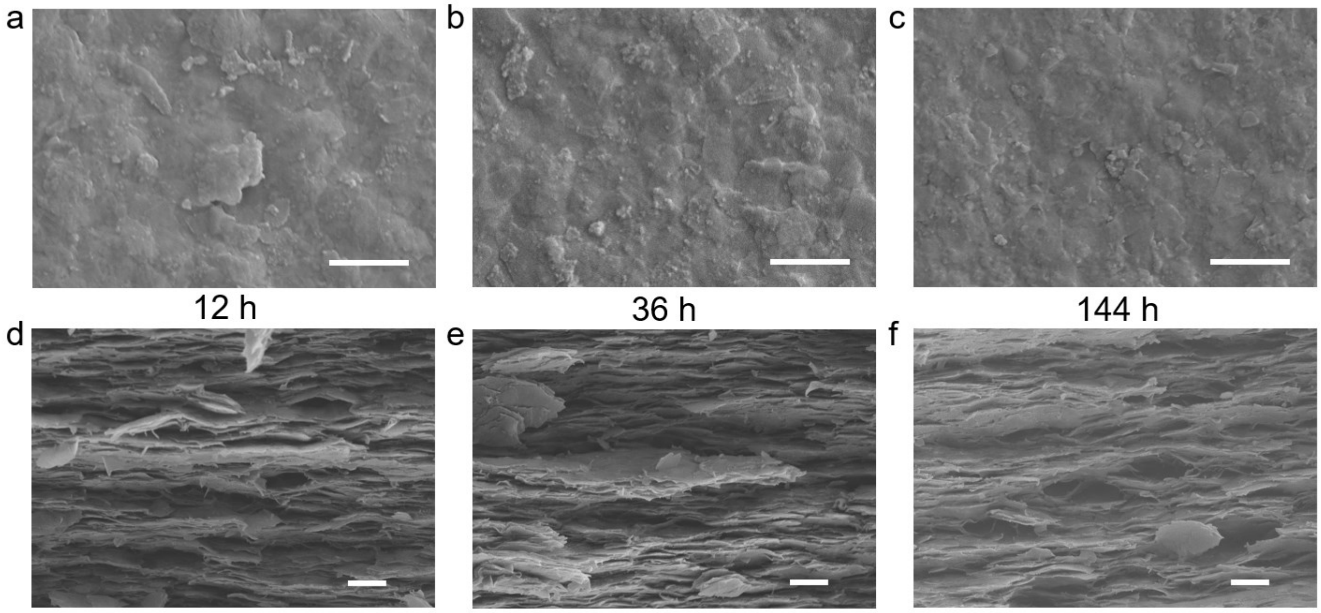

Supplementary Figure 16 | Microstructure of polymeric mica film after UV irradiation. a-f, SEM images of surfaces (**a-c**) and cross sections (**d-f**) of polymeric mica film after 12 h, 36 h, and 144 h UV irradiation, respectively. *Scale bars*, 1 µm.

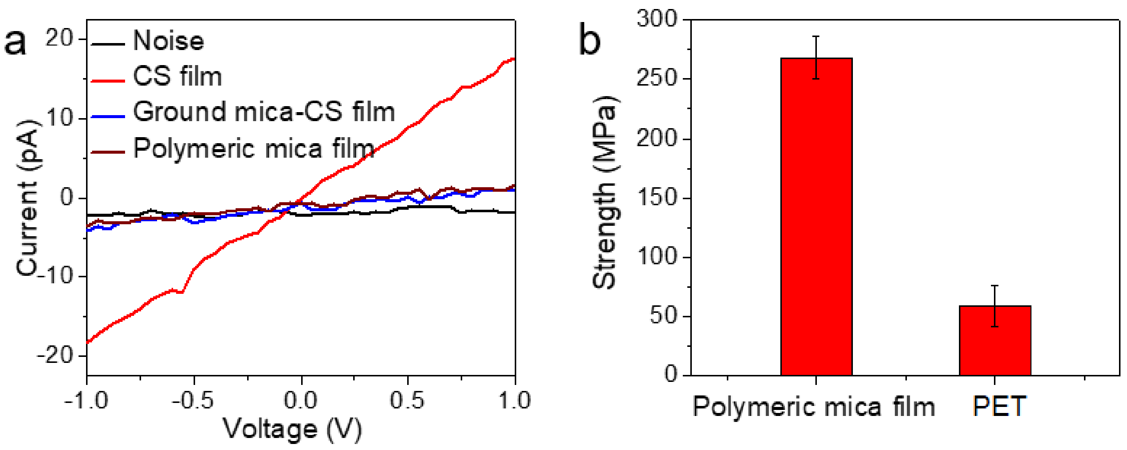

Supplementary Figure 17 | Electrical and mechanical characterization of polymeric mica, ground mica-CS, and PET films. **a**, I-V curves of pure CS, 60 wt.% ground mica-CS, and 60 wt.% polymeric mica film. **b**, Tensile strength of 60 wt.% polymeric mica and PET films. All the error bars represent the s.d. of at least five replicate measurements.

Supplementary Table 1 | Elemental composition of ground mica and eMica nanosheets measured by XPS.

	Element composition (wt.%)				
**Element**	Si	Al	O	K	Mg
**Ground mica**	18.1	13.5	60.9	3.5	2.0
**eMica nanosheets**	18.1	13.3	61.9	3.0	2.2

Supplementary Table 2 | Comparison of the production rate of eMica nanosheets with other exfoliated thin nanosheets in previous works.

Sample	Method	Volume	Production rate	Ref
Graphene	Shear exfoliation	300 L	5.3 g h^{-1}	4
Graphene	Intercalation with K (glovebox)	/	0.9 g h^{-1}	5
GO	produced by modified Hummer's method	2 L	0.6 g h^{-1}	6
Graphene	Interlayer catalytic exfoliation with $FeCl_3$ and H_2O_2 (glovebox)	/	0.4 g h^{-1}	7
Graphene	Exfoliation by chlorosulfonic acid and H_2O_2	/	0.25 g h^{-1}	8
Graphene	Electrochemical charging and Li intercalation of a graphite electrode	/	0.12 g h^{-1}	9
MoS_2	Ultrasonication in an aqueous surfactant solution	1 L	0.39 g h^{-1}	10
BN	Ultrasound in alcohol	/	0.4 g h^{-1}	11
WS_2	Ultrasonication in aqueous solutions of biopolymers or surfactants	20 mL	0.028 g h^{-1}	12
$CoSe_2$	Ultrasonication in aqueous solutions	10 mL	/	13
Sb	Ultrasonication in isopropyl alcohol	20 mL	0.00013 g h^{-1}	14
MoS_2	Ultrasonication in ethanol/water solutions	10 mL	0.012 g h^{-1}	15
Ta_2NiS_5	Sonication in water	/	/	16
MTM	Stirring in water	1 L	0.018 g h^{-1}	17
Mica	Microwave irradiated with sonication	/	/	18
**eMica**	**Ultrasonication in ethanol**	**500 mL**	**1 g h^{-1}**	**This work**

Supplementary Table 3 | Zeta potential values of DIW dispersions of ground mica, eMica nanosheets, CS-eMica nanosheets, and PEI-eMica nanosheets.

Sample	Zeta potential (mV)	Standard deviation (s. d.)
**Ground mica**	-7.87	1.81
**eMica nanosheets**	-32.23	0.87
**CS-eMica nanosheets**	17.2	0.93
**PEI-eMica nanosheets**	44.20	0.98

Supplementary Table 4 | Comparison of mechanical and optical performances of polymeric mica film with other nanoclay-based biomimetic films.

Materials	Strength (MPa)	400-800 nm	200-400 nm	Thickness (μm)	Nanoclay content (wt.%)	Ref
MTM/PVA	248	40-70%	10%-40% (300-400 nm)	20	70	19
MTM/PVA	219	18%-68%	/	9	70	17
MTM/CMC	319	60-80%	20-60% (300-400 nm)	40-50	20	20
MTM/NFC/PVA	302	68-88%	/	9	70	21
EG-UPy-NHT	265	84-88%	/	25	70	22
NTS/PVA	215	50-72%	/	25	76	23
SUM/PVA	150	86-90%	/	25	50	23
LAP/PVA	81	95-98%	/	25	45	23
MTM/CS	100	50-80%	20-50% (300-400 nm)	/	65	24
MTM/CS/NFC	132	/	/	/	91	25
LDH/CS	160	22-21%	0-22%	/	/	26
MTM/PVA	315	88-92%	/	/	50	27
LDH/PVA	195	/	/	/	16	28
MTM/PDDA	151	/	/	/	70	29
MMT/PDDA/dGMP	149	/	/	/	77	30
MTM/CMC	245	/	/	/	20	31
Polymeric mica film	259	37.8-65.5%	0-37.8%	25	60	This work
**Polymeric mica film**	**259**	**57.5-84.6%**	**0-57.5%**	**5**	**60**	**This work**

Supplementary References

1. Ding, H., Wang, Y. B., Liang, Y. & Qin, F. X. Preparation and characterization of cetyl trimethylammonium intercalated sericite. *Adv. Mater. Sci. Eng.* **2**, 480138 (2014).
2. Gao, H. L. *et al.* Super-elastic and fatigue resistant carbon material with lamellar multi-arch microstructure. *Nat. Commun.* **7**, 12920 (2016).
3. Tezcan, F., Günister, E., Ozen, G. & Erim, F. B. Biocomposite films based on alginate and organically modified clay. *Int. J Biol. Macromol.* **50**, 1165-1168 (2012).
4. Paton, K. R. *et al.* Scalable production of large quantities of defect-free few-layer graphene by shear exfoliation in liquids. *Nat. Mater.* **13**, 624-630 (2014).
5. Viculis, L. M., Mack, J. J., Mayer, O. M., Hahn, H. T. & Kaner, R. B. Intercalation and exfoliation routes to graphite nanoplatelets. *J. Mater. Chem.* **15**, 974-978 (2005).
6. Liao, K. H. *et al.* Aqueous only route toward graphene from graphite oxide. *ACS Nano* **5**, 1253-1258 (2011).
7. Geng, X. *et al.* Interlayer catalytic exfoliation realizing scalable production of large-size pristine few-layer graphene. *Sci. Rep.* **3**, 1134 (2013).
8. Lu, W. *et al.* High-yield, large-scale production of few-layer graphene flakes within seconds: using chlorosulfonic acid and H_2O_2 as exfoliating agents. *J. Mater. Chem.* **22**, 8775-8777 (2012).
9. Wang, J., Manga, K. K., Bao, Q. & Loh, K. P. High-yield synthesis of few-layer graphene flakes through electrochemical expansion of graphite in propylene carbonate electrolyte. *J. Am. Chem. Soc.* **133**, 8888-8891 (2011).
10. Smith, R. J. *et al.* Large- scale exfoliation of inorganic layered compounds in aqueous surfactant solutions. *Adv. Mater.* **23**, 3944-3948 (2011).
11. Zhu, W. S. *et al.* Controlled gas exfoliation of boron nitride into few-layered nanosheets. *Angew. Chem. Int. Ed.* **55**, 10766-10770 (2016).
12. Zong, L., Li, M. & Li, C. Bioinspired coupling of inorganic layered nanomaterials with marine polysaccharides for efficient aqueous exfoliation and smart actuating hybrids. *Adv. Mater.* **29**, 1604691 (2017).
13. Liu, Y. *et al.* Low overpotential in vacancy-rich ultrathin $CoSe_2$ nanosheets for water oxidation. *J. Am. Chem. Soc.* **136**, 15670-15675 (2014).
14. Gu, J. *et al.* Liquid- phase exfoliated metallic antimony nanosheets toward high volumetric sodium storage. *Adv. Energy Mater.* **7**, 1700447 (2017).

15. Fan, X. B. *et al.* Controlled exfoliation of MoS_2 crystals into trilayer nanosheets. *J. Am. Chem. Soc.* **138**, 5143-5149 (2016).
16. Tan, C. *et al.* High-yield exfoliation of ultrathin two-dimensional ternary chalcogenide nanosheets for highly sensitive and selective fluorescence DNA sensors. *J. Am. Chem. Soc.* **137**, 10430-10436 (2015).
17. Wang, J. F., Cheng, Q. F., Lin, L., Chen, L. F. & Jiang, L. Understanding the relationship of performance with nanofiller content in the biomimetic layered nanocomposites. *Nanoscale* **5**, 6356-6362 (2013).
18. Van Khai, T. *et al.* Synthesis and characterization of single-and few-layer mica nanosheets by the microwave-assisted solvothermal approach. *Nanotechnology* **24**, 145602 (2013).
19. Walther, A. *et al.* Large-area, lightweight and thick biomimetic composites with superior material properties via fast, economic, and green pathways. *Nano Lett.* **10**, 2742-2748 (2010).
20. Das, P. *et al.* Facile access to large-scale, self-assembled, nacre-inspired, high-performance materials with tunable nanoscale periodicities. *ACS Appl. Mater. Interfaces* **5**, 3738-3747 (2013).
21. Wang, J. F., Cheng, Q. F., Lin, L. & Jiang, L. Synergistic toughening of bioinspired poly(vinyl alcohol)-clay-nanofibrillar cellulose artificial nacre. *ACS Nano* **8**, 2739-2745 (2014).
22. Zhu, B. *et al.* Hierarchical nacre mimetics with synergistic mechanical properties by control of molecular Interactions in self- healing polymers. *Angew. Chem. Int. Ed.* **54**, 8653-8657 (2015).
23. Das, P. *et al.* Nacre-mimetics with synthetic nanoclays up to ultrahigh aspect ratios. *Nat. Commun.* **6**, 5967 (2015).
24. Yao, H. B., Tan, Z. H., Fang, H. Y. & Yu, S. H. Artificial nacre-like bionanocomposite films from the self-assembly of chitosan-montmorillonite hybrid building blocks. *Angew. Chem. Int. Ed.* **49**, 10127-10131 (2010).
25. Liu, A. & Berglund, L. A. Clay nanopaper composites of nacre-like structure based on montmorrilonite and cellulose nanofibers-improvements due to chitosan addition. *Carbohydr. Polym.* **87**, 53-60 (2012).
26. Yao, H. B., Fang, H. Y., Tan, Z. H., Wu, L. H. & Yu, S. H. Biologically inspired, strong, sransparent, and functional layered organic-inorganic hybrid films. *Angew. Chem. Int. Ed.* **49**, 2140-2145 (2010).
27. Ding, F. *et al.* Biomimetic nanocoatings with exceptional mechanical, barrier, and flame-retardant properties from large-scale one-step coassembly. *Sci. Adv.* **3**, e1701212 (2017).

28. Han, J. *et al.* Biomimetic design and assembly of organic-inorganic composite films with simultaneously enhanced strength and toughness. *Chem. Commun.* **47**, 5274-5276 (2011).
29. Walther, A. *et al.* Supramolecular control of stiffness and strength in lightweight high- performance nacre- mimetic paper with fire- shielding properties. *Angew. Chem. Int. Ed.* **49**, 6448-6453 (2010).
30. Martikainen, L., Walther, A., Seitsonen, J., Berglund, L. & Ikkala, O. Deoxyguanosine phosphate mediated sacrificial bonds promote synergistic mechanical properties in nacre-mimetic nanocomposites. *Biomacromolecules* **14**, 2531-2535 (2013).
31. Das, P. & Walther, A. Ionic supramolecular bonds preserve mechanical properties and enable synergetic performance at high humidity in water-borne, self-assembled nacre-mimetics. *Nanoscale* **5**, 9348-9356 (2013).
